# Nanomechanical *In Situ* Monitoring of Proteolysis of Peptide by Cathepsin B

**DOI:** 10.1371/journal.pone.0006248

**Published:** 2009-07-16

**Authors:** Taeyun Kwon, Jinsung Park, Jaemoon Yang, Dae Sung Yoon, Sungsoo Na, Chang-Wan Kim, Jin-Suck Suh, Yong-Min Huh, Seungjoo Haam, Kilho Eom

**Affiliations:** 1 Research Institute for Engineering Technology, Korea University, Seoul, Republic of Korea; 2 Department of Mechanical Engineering, Korea University, Seoul, Republic of Korea; 3 Department of Radiology, College of Medicine, Yonsei University, Seoul, Republic of Korea; 4 Department of Biomedical Engineering, Yonsei University, Kangwon-do, Republic of Korea; 5 Department of Mechanical Engineering, Konkuk University, Seoul, Republic of Korea; 6 Department of Chemical and Biomolecular Engineering, Yonsei University, Seoul, Republic of Korea; Dalhousie University, Canada

## Abstract

Characterization and control of proteolysis of peptides by specific cellular protease is *a priori* requisite for effective drug discovery. Here, we report the nanomechanical, *in situ* monitoring of proteolysis of peptide chain attributed to protease (Cathepsin B) by using a resonant nanomechanical microcantilever immersed in a liquid. Specifically, the detection is based on measurement of resonant frequency shift arising from proteolysis of peptides (leading to decrease of cantilever's overall mass, and consequently, increases in the resonance). It is shown that resonant microcantilever enables the quantification of proteolysis efficacy with respect to protease concentration. Remarkably, the nanomechanical, *in situ* monitoring of proteolysis allows us to gain insight into the kinetics of proteolysis of peptides, which is well depicted by Langmuir kinetic model. This implies that nanomechanical biosensor enables the characterization of specific cellular protease such as its kinetics.

## Introduction

Over-manifestation of the cellular protease is the kernel factor for genesis of various human body disorders. The development mechanism of a protease and their proteolysis of specific peptide (or protein) as substrate have played a pivotal role on genesis of inflammatory disease and outbreaks of cancer and their metastasis by sudden change of physiological condition and immune system [1]. Recently, synthetic polymers conjugated to drug (or specific molecules) via peptide linker chain have been employed as a targeted drug carrier [2]–[4]. When the drug carrier encounters the specific cancer cells where specific protease is contained, the effective release of drug is attributed to proteolysis of peptide linker chain, and released drug attacks tumor mass. Thus, comprehension and control of proteolysis (i.e. peptide-protease interaction) can provide the important information for drug discovery [5].

Nanomechanical devices such as microcantilevers have enabled the characterization of interactions between various biological molecules such as DNA hybridization [6]–[9], protein antigen-antibody binding [10], RNA-protein interaction [11], peptide-drug interaction [12], and ligand-binding on membrane protein [13], [14]. Label-free detection of such interactions is typically based on the measurement of cantilever bending deflection change arising from such molecular interactions. It is a simple, straightforward principle, that is, transduction of chemical interaction on cantilever surface into a cantilever bending deflection change, which is well described by Stoney's equation [15]. However, the relationship between surface stress and molecular interaction on the surface is not straightforward, albeit recently there have been attempts [16], [17] to theoretically make a connection between surface stress and molecular interactions. Moreover, it is difficult to quantify how many molecules are involved in molecular interactions [14].

Recently, instead of label-free detection using cantilever bending deflection, there has been an alternative in the label-free detection based on evaluation of cantilever's resonant frequency shift driven by molecular interaction on a cantilever surface. Unlike the former case (using bending deflection), resonant microcantilever enables us to quantify the amount of molecules involved in molecular interactions [14], [18]–[20]. It has been remarkably reported that resonant microcantilevers enable the highly sensitive detection of chemical molecules even at atomistic resolution [21], [22], which is ascribed to the scaling down leading to increase of dynamic-frequency range. The relationship between molecular binding and resonant frequency shift has been suggested based on continuum elastic model [18], [19], [22]. Specifically, as long as cantilever thickness is relatively larger than that of molecular monolayer on the cantilever surface, the resonant frequency shift is linearly proportional to the total mass of molecules involved in molecular binding (or interaction) on the surface [22]. In case of thin cantilever (whose thickness is comparable to that of molecular monolayer), the relationship between resonant frequency shift and molecular interaction is unclear and complex because of several possible effects such as surface stress [23], and elastic properties of molecular monolayer [24], [25]. In recent years, resonant microcantilevers have allowed for label-free detection of DNA molecules (even in a single-molecule level) [26], proteins [19], virus [25], and/or chemicals [21] typically in dry air or vacuum. On the other hand, such detection based on resonant frequency shift measured in dry air or vacuum restricts our understanding of biochemical reactions in fluid with a real-time, indicative for kinetics of biochemical reactions [20], [27]. For gaining insight into such kinetics, there has been a recent attempt [20] to develop the microcantilever to exhibit the relatively high quality factor, which will lead to *in situ* detection of biochemical reaction, and their related kinetics.

In this study, we report the nanomechanical, *in situ* monitoring of proteolysis of peptide, which is usually employed as a linker molecule for drug carrier, attributed to protease (Cathepsin B, CTSB) using resonant microcantilever immersed in buffer solution. Specifically, resonant microcantilever allows us to quantify the amount of peptide chains involved in proteolysis, and consequently, proteolysis efficiency with respect to enzyme concentration. Such proteolysis of peptide chain is ensured from conventional experiments such as MALDI-TOF (Matrix-Assisted Laser Desorption/Ionization – Time Of Flight) mass spectrometry as well as AFM imaging of functionalized cantilever surface with or without exposure to protease. Moreover, it is remarkably shown that resonant microcantilever immersed in a liquid enables the characterization of proteolysis such as its kinetics that is well dictated by Langmuir kinetic model.

## Materials and Methods

### Synthesis of Carboxylated Polyethylene Glycol (PEG-COOH)

The synthetic carboxylated polyethylene glycol (PEG-COOH) [28] is presented in Figure S1.A in Supporting Information. Polyethylene glycol (PEG-OH; 5,000 Da; Fluka) was first modified by anhydride-acylation using succinic anhydride. PEG-OH (0.4 mmol) were dissolved in 1, 4-dioxane (100 mL) and stirred for 24 hours at room temperature under nitrogen ambient. After solvent evaporation, the white powders were obtained and then dissolved in chloroform to remove un-reactants by filtration (pore size: 200 nm, ADVANTEC®). The transparency solution was precipitated against excess cold diethyl ether with a drop-wise manner. The precipitates (PEG-COOH) were dried under vacuum and stored until later use. We confirmed the characteristic band of carboxylated PEG (PEG-COOH) using the FT-IR spectra (see Figure S1.B in Supporting Information). The anhydride group of succinic anhydride (1,783 and 1,861 cm^−1^) was converted into carboxyl group (1,732 cm^−1^) after esterification of the hydroxyl group of PEG by the ring opening process of succinic anhydride. A Biflex III (Bruker Daltonics) time-of-flight mass spectrometer equipped with a 337-nm nitrogen laser was used to record MALDI-TOF mass spectra of the sample (see Figure S2 in Supporting Information).

### Preparation of PEGylated GFLG (PEG-GFLG)

Tetrapeptide GFLG (GlyPheLysGly) [29] was obtained from Peptron, Inc. (Korea). N-terminal of GFLG was conjugated with carboxyl group of PEG-COOH by esterification reaction. PEG-COOH (20 µmol), N-hydroxysuccinimide (NHS, 40 µmol), and 1-ethyl-3-(3-dimethyl-. Aminopropyl)-carbodiimide (EDC, 40 µmol) were dissolved in phosphate buffered saline (PBS; 1 mL, pH 7.4, 10 mM). After incubation of the mixture for 6 hours at room temperature, the products were purified by excess ethanol. For preparation of PEGylated GFLG (PEG-GFLG), succinimidyl PEG (10 µmol) was subsequently conjugated with N-terminal of GFLG (10 µmol) for 30 minutes at 4°C. Residual reactants were removed by filtration (MWCO: 3,000 Da; AMICON Ultra).

### Preparation of Microcantilever

We have utilized the microcantilever (RTSEP – Tap300, Metrology Probe, Veeco, Santa Barbara, CA), whose dimension is given as 35×4×125 µm^3^ (width×thickness×length) with a force constant of ∼40 N/m. The fundamental resonance of such a microcantilever operated in dry air is within the range of 300±100 kHz. This is consistent with experimentally measured resonance of our microcantilever such as *ω*_0_ = 269.3 kHz (for Cantilever 1 in Table 1). For nanomechanical detection of proteolysis, the peptide chains (i.e. PEG-GFLG) were immobilized on the aminated cantilever surface as follows. The cantilever surface was functionalized by amine such that surface is chemically modified by 3-aminopropyltrimethoxysilane (100 µL; Sigma, St Louis, MO) in 20 mL water at 80°C for 24 hours. After chemical reaction, aminated surface was purified by excessive water and ethanol. PEG-GFLG was immobilized by using EDC/NHS. In detail, PEG-GFLG (10 µmol), NHS (40 µmol), and EDC (40 µmol) were dissolved in phosphate buffered saline (PBS; 20 mL, pH 7.4, 10 mM). After incubation of the mixture with aminated cantilever for 24 hours at 25°C, the products were purified by excess buffer solution.

**Table 1:** Resonances of bare cantilevers, cantilevers after peptide immobilization, and such peptide-immobilized cantilevers after exposure to protease with protease concentrations of 0.28 µM, 1.12 µM, 1.53 µM, and 1.61 µM, respectively, were measured in dry air or liquid (only for Cantilever 1).

	[CTSB] (µM)	*ω*_0_ (kHz)	Δ*ω_I_* (kHz)	Δ*ω_P_* (kHz)	Δ*m_I_* (ng)	Δ*m_P_* (ng)
**Cantilever 1 (in dry air)**	0.28	269.3	+17.6	−1.2	5.33	0.44
**Cantilever 1 (in liquid)**	0.28	116	+6.5	−2.1		
**Cantilever 2 (in dry air)**	1.12	265.29	+16.41	−5.32	5.04	1.96
**Cantilever 3 (in dry air)**	1.53	353.02	+13.11	−8.45	3.03	2.18
**Cantilever 4 (in dry air)**	1.61	317.09	+11.51	−9.46	2.96	2.71

From Eq. (3), the total amount of immobilized peptides and cleft peptides, respectively, were also computed. Here, *ω*_0_, Δ*ω_I_*, Δ*ω_P_*, Δ*m_I_*, and Δ*m_P_* indicate the resonant frequency of a bare cantilever, the resonant frequency shift due to peptide immobilization, the resonant frequency shift induced by proteolysis by protease, the total mass of immobilized peptides on cantilever's surface, and the total amount of cleft peptides due to protease with a given protease concentration [CTSB], respectively. The positive sign in the resonant frequency shift indicates the increase of the resonance, while the negative sign shows the decrease of the resonance.

### Measurement of the Resonance

The resonant frequency of a microcantilever is measured from Nanoscope V controller (Picoforce, Veeco, Santa Barbara, CA). Specifically, the software Nanoscope v7.0 (Veeco, Santa Barbara, CA) generates the resonance-amplitude curve, in which the sharp peak indicates the fundamental resonance. The resonance measured from Nanoscope V controller is confirmed by classical elasticity theory (for details, see Results and Discussions). For *in situ* monitoring of proteolysis, a microcantilever functionalized by PEG-GFLG was mounted on an O-ring liquid cell with a volume of ∼50 µL. First, the resonance of such a microcantilever was measured in PBS solution. At 25°C, then, CTSB solution with different concentrations (0.28 µM, 0.56 µM, 0.84 µM) was injected into a liquid cell. Subsequently, the *in situ* resonance of a cantilever in buffer solution was monitored for every 20 minutes after injection of CTSB solution over 15 hours. However, it should be kept in mind that, for quantification of mass of cleft peptides by CTSB, the resonant frequency shift has to be measured in dry air. It is attributed to fact that the resonant frequency shift measured in liquid due to protease is ascribed to total amount of cleft peptides as well as the hydrodynamic loading change due to hydrophilicity change during enzymatic cleavage [19]. In other words, hydrodynamic loading change during the enzymatic cleavage can be also estimated from the resonant frequency shift measured in liquid, since the total amount of cleft peptides is directly evaluated from the resonant frequency shift measured in dry air. For measurement of resonant frequency shift in dry air due to enzymatic cleavage, we have to dry up the cantilever which was utilized for *in situ* bioassay. Such a cantilever was dried in the jell-pad in a desiccator for a few hours at room temperature. Then the resonance of such a cantilever, on which the peptide chains are likely to be cleft by CTSB, is measured, and consequently, so is the resonant frequency shift in dry air due to proteolysis driven by CTSB.

## Results and Discussion

### Characterization of Resonance Behavior of Microcantilever

Classical elastic continuum model of a cantilever provides the resonance of a cantilever operated in a dry air in the form of [18], [19]

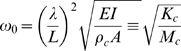
(1)


where *L*, *EI*, *A*, and *ρ_c_* are a cantilever's length, bending rigidity, cross-sectional area, and density, respectively, and a parameter *λ* satisfies the transcendental equation such as cos*λ*cosh*λ* + 1 = 0. Further, *M_c_* and *K_c_* represent the cantilever's effective mass and effective stiffness, respectively, given by *M_c_* = *ρ_c_A* and *K* = *λ* ^4^ *EI*/*L* ^3^. Such an elastic continuum model predicts the fundamental resonance of our cantilever in dry air is given by 275 kHz, consistent with experimentally measured resonance of 269. 3 kHz. Here, the dimension of a cantilever is given by *L*×*w_c_*×*t_c_* (length×width×thickness), where *L* = 125 µm, *w_c_* = 30 µm, and *t_c_* = 3 µm. For *in situ* monitoring of molecular interaction in buffer solution, the resonance behavior of a cantilever in a fluid has to be characterized. Once a cantilever is immersed in a liquid, the hydrodynamic loading plays a significant role on the resonance behavior of a cantilever. Specifically, the resonance behavior of a microcantilever immersed in a fluid is given by *ω_L_* = *ω*_0_ *θ* ^1/2^ [19], [30], where *ω*_0_ represents the fundamental resonance measured in dry air, and a parameter *θ* is given as *θ* = *M_c_*/(*M_c_* + *M_h_*) with *M_h_* being a hydrodynamic loading. Here, the hydrodynamic loading *M_h_* can be estimated from a relation of [30]

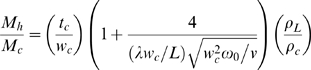
(2)


Here, *ν* and *ρ_L_* indicate the kinetic viscosity and the density of a liquid, respectively. The elastic continuum model predicts the hydrodynamic loading of *M_h_* = 112 ng, and consequently, the resonance of a cantilever immersed in a fluid as *ω_L_* = 119. 7 kHz. This is consistent with experimentally measured resonance in a fluid such as *ω_L_* = 113.6 kHz.

### Detection Principle Based on Resonant Frequency Shift

It is well known that resonant frequency shift is mainly attributed to the mass of molecules involved in molecular interactions (e.g. proteolysis) rather than any other effects such as surface elasticity of molecular monolayer (e.g. PEG-GFLG immobilized on the cantilever surface). For instance, if the thickness of a cantilever is comparable to that of molecular monolayer, then surface effect such as surface stress [23] and surface elastic properties [24], [25] play a role on the resonant frequency shift due to molecular interactions. Since the thickness of our cantilever is much larger than that of molecular monolayer, the resonant frequency shift is related to mass of molecules. As stated earlier in Methods and Materials, we have considered the resonant frequency shift measured in dry air due to protease in order to quantify the amount of cleft peptides. It is because the resonant frequency shift measured in buffer solution during the proteolysis by protease is attributed to not only the mass of cleft peptides but also the hydrodynamic loading change originated from hydrophilicty change [19]. It is very straightforward that the resonant frequency shift, measured in dry air, due to molecular interaction is directly related to mass of molecules such as [18], [19]

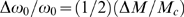
(3)


where Δ*ω*_0_ is the resonant frequency shift measured in normal air, and Δ*M* is the total mass of molecules involved in molecular interactions. However, if resonant frequency shift induced by molecular interactions is measured in a liquid, then the hydrodynamic loading does also play a role on the resonant frequency shift. Specifically, the resonant frequency shift, which is measured in buffer solution, induced by molecular interactions (here, proteolysis) is attributed to not only the mass of molecules involved in molecular interactions but also the hydrodynamic loading effect arising from the hydrophilicity change during the interactions [19].
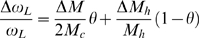
(4)


**Figure 1:**
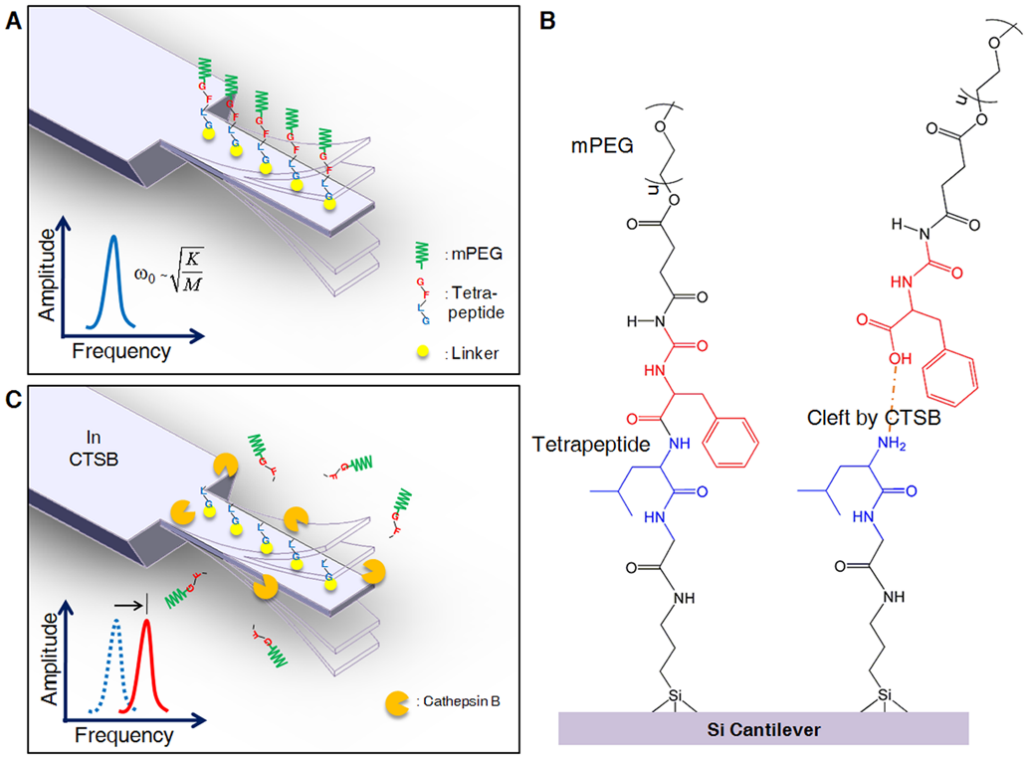
Schematic illustration of nanomechanical, *in situ* monitoring of proteolysis of peptide chains on the surface by using resonant microcantilever immersed in a liquid. (A) A microcantilever was functionalized by peptide chains (PEGylated GFLG) through aminated cantilever surface. The fundamental resonance of a cantilever is given by *ω* = (*K*/*M_c_*)^1/2^, where *K* is the overall stiffness of a cantilever, and *M_c_* is the overall mass of a cantilever. (B) Chemical structure of PEGylated GFLG (GlyPheLysGly) chains on a cantilever and proteolyzed peptides by protease (i.e. PEG-GF and LG sequence immobilized on a cantilever). (C) When GFLG peptides immobilized on a cantilever was exposed to protease (CTSB) in acidic medium, catalytic Cys25 and His159 of CTSB induces the successful cystein protease of GFLG, leading to proteolysis of GFLG. Such proteolysis phenomenon reduces the overall mass of a cantilever, and consequently, the increase of the fundamental resonance.

Here, Δ*ω_L_* is the resonant frequency shift, which is measured in buffer solution, driven by molecular interaction, and Δ*M_h_* is the change of hydrodynamic loading, which arises from hydrophilicity change during the interaction. Schematic illustration in Figure 1 demonstrates the nanomechanical detection principle such as transduction of proteolysis into the resonant frequency shift. In detail, proteolysis of GFLG conjugated to PEG, which is immobilized on the surface of microcantilever, driven by lysosomal cystein protease from Cathepsin B (CTSB) [31], [32] induces the decrease of cantilever's overall mass leading to increase of resonance of a cantilever (see Figure 1). This straightforward detection principle allows us to quantify the total mass (or number) of molecules involved in molecular interactions. In other words, the total mass of cleft peptide chains due to proteolysis of GFLG driven by CTSB can be quantified based on the resonant frequency shift related to mass change.

### Label-Free Detection of Proteolysis and Proteolysis Efficiency

For nanomechanical, label-free detection of proteolysis, we have functionalized the microcantilever surface with amine group and GFLG was modified using PEG molecules (see Methods and Materials). Specifically, in acidic medium (PBS solution with pH 5 and 10 mM), catalytic Cys25 and His159 of CTSB induces the successful proteolysis of GFLG [32]. Here, PEG molecule is hydrophilic so that water molecules allow PEG molecules to be a one-dimensional structure [33], which enables CTSB to easily attack the GFLG. It should be noted that PEG molecules were conjugated to GFLG in order to easily detect the proteolysis of GFLG based on the frequency shift due to mass of PEG (for details, see below). For label-free detection of such a proteolysis, we have employed the silicon cantilever functionalized by PEG-GFLG (for details, see Methods and Materials). Herein, PEG-GFLG chains were immobilized by immersing an aminated cantilever into buffer solution (PBS, pH 5) which contains the peptides with concentration of 10 mM. In order to estimate the total mass of immobilized peptide chains, we have measured the resonant frequencies of cantilever with or without immobilization of peptides in dry air (see Figure S3 in Supporting Information), since we have the straightforward relationship between frequency shift measured in dry air and total mass of immobilized peptide chains. It is shown that the frequency shift, which was measured in dry air, due to peptide immobilization provides the total mass of immobilized peptides as Δ*m_I_* = 4.56 ng (for Cantilever 1 in Table 1).

Now, let us consider the label-free detection of proteolysis of GFLG using resonant cantilever functionalized by PEG-GFLG chains. In order to confirm the specific proteolysis of GFLG by CTSB, we have considered the control experiments. First, we have taken into account the resonance behavior of a cantilever, which is functionalized by GFLG-PEG chains, in buffer solution. It is shown that resonant frequency of such a functionalized cantilever is constant with respect to time, which indicates that the resonance of our cantilever is stable in buffer solution (see Figure S4.A in Supporting Information). Second, we have considered the resonance behavior of a bare cantilever in exposure to CTSB in buffer solution. It is shown that injection of CTSB does not induce any resonant frequency shift of a bare cantilever, which indicates that non-specific binding onto a cantilever is unlikely to occur (see Figure S4.B in Supporting Information). Finally, we have taken into account the resonance behavior of a cantilever functionalized by PEG (without conjugation to GFLG) in response to injection of CTSB in buffer solution. It is shown that injection of CTSB does not drive any resonant frequency shift of a cantilever functionalized by PEG (see Figure S4.C in Supporting Information). This confirms the specific proteolysis of GFLG by CTSB.

**Figure 2:**
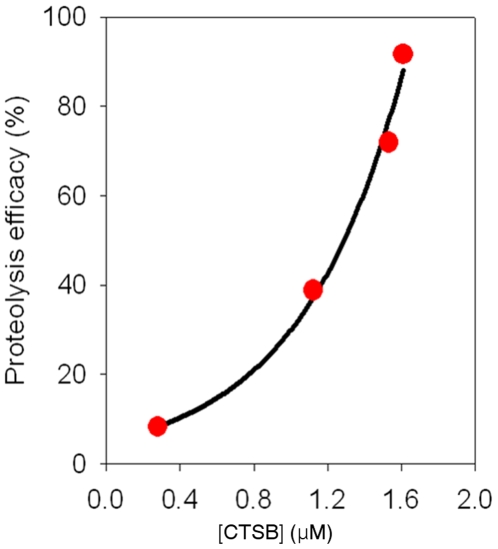
Relationship between proteolysis efficiency, *r*, and CTSB (Cathepsin B) concentration, [CTSB] is shown. Here, proteolysis efficiency, *r*, is defined as *r* = Δ*m_P_*/Δ*m_I_*, where Δ*m_P_* and Δ*m_I_* represent the total mass of cleft peptides and immobilized GFLG peptides on the cantilever surface, respectively. The mass of immobilized peptides is measured from the resonance frequency difference between a bare cantilever and cantilever functionalized by peptides, while the mass of cleft peptides driven by protease is evaluated from the resonance difference between cantilever functionalized by peptides and such a cantilever in exposure to CTSB. The proteolysis efficiency is exponentially proportional to the CTSB concentration in buffer solution. Here, the measurement of resonant frequency shifts due to proteolysis based on 4 different cantilevers (e.g. Table 1) was implemented in dry air.

We consider the label-free detection of specific proteolysis based on the resonant frequency shift, which was measured in dry air, induced by proteolysis of GFLG on a cantilever. Here, in order to estimate the exact amount of cleft peptides due to CTSB, we consider the resonance, which was evaluated in dry air, of a cantilever before and after the injection of CTSB (for details, see *Measurement of the Resonance* and *Detection Principle Based on Resonant Frequency Shift*). It should be recognized that measurement of resonant frequency shift due to molecular interaction in buffer solution may not allow for direct computation of molecules involved in such interaction because of hydrodynamic loading effect coupled to resonant frequency shift measured in buffer solution [19]. As shown in Table 1, we have employed four different cantilevers for measuring the resonant frequency shifts due to proteolysis with different protease concentrations. Table 1 shows the resonant frequencies of four bare cantilevers (before peptide immobilization), the resonant frequency shifts measured in dry air due to peptide immobilization, and the resonant frequency shifts evaluated in dry air due to proteolysis driven by protease with different protease concentrations (i.e. 0.28 µM, 1.12 µM, 1.53 µM, and 1.61 µM). From Eq. (3), the total amount of immobilized peptides on a cantilever and the amount of cleft peptides were obtained as shown in Table 1. In addition, based on Cantilever 1 in Table 1, we have computed the hydrodynamic loading change during the proteolysis from the resonant frequency shifts measured in dry air and liquid, respectively. From Eqs. (3) and (4), it is shown that hydrodynamic loading change during the proteolysis is given as Δ*M_h_* = 0.502 ng. This indicates that the amount of hydrodynamic loading change is comparable to that of cleft peptides, i.e. Δ*M_h_* ≈ Δ*m_P_*, where Δ*m_P_* represents the total mass of cleft peptides. Then, we have introduced the proteolysis efficiency, *r*, such as the ratio of the amount of cleft peptides to that of immobilized peptides, i.e. *r* = Δ*m_P_*/Δ*m_I_*, where Δ*m_I_* represent the total mass immobilized peptides. Here, the total mass of cleft peptides, Δ*m_P_*, can be obtained from the resonant frequency shift, Δω_P_^*^, which was measured in dry air, due to proteolysis. The relation between frequency shift (measured in dry air), Δ*ω_P_* ^*^, due to proteolysis (protease) and total mass of cleft peptides, Δ*m_P_*, is given by Δ*ω_P_* ^*^ = (*ω_I_*/2)[ Δ*m_P_*/(*M_c_* + Δ*m_I_*)], where *ω_I_* is the resonant frequency of a cantilever, which was functionalized by peptides, in dry air, and *M_c_* is the mass of a bare cantilever. Figure 2 shows the proteolysis efficiency, *r*, with respect to protease (CTSB) concentrations, [CTSB]. This indicates that resonant microcantilever is capable of quantification of cleft peptides attributed to protease, and consequently, proteolysis efficiency. Further, the proteolysis of PEG-GFLG on the cantilever surface is also confirmed by conventional experimental methods such as MALDI-TOF mass spectrometry and AFM imaging. MALDI-TOF mass spectrometry suggests that total molecular mass of a single GFLG-PEG chain is 8.64 zg (zepto-gram = 10^−21^ g), where carboxylated PEG exhibits the molecular mass of 8.07 zg, and that the total mass of a single proteolyzed chain is 8.30 zg (see Figure S2 in Supporting Information). This confirms that CTSB specifically cleaves the GFLG chain rather than PEG chain. Moreover, we have considered the surface profiles of three different cantilever surfaces – the surface of a bare cantilever, the cantilever surface where PEG-GFLG chains were functionalized, and the surface of such a functionalized cantilever after exposure to CTSB, respectively (for details, see Supporting Information). The surface profiles of three different cantilever surfaces obtained from AFM imaging confirms the proteolysis event on the microcantilever surface due to CTSB (see also Figure S5 in Supporting Information).

### In Situ Monitoring and Kinetics of Proteolysis

**Figure 3:**
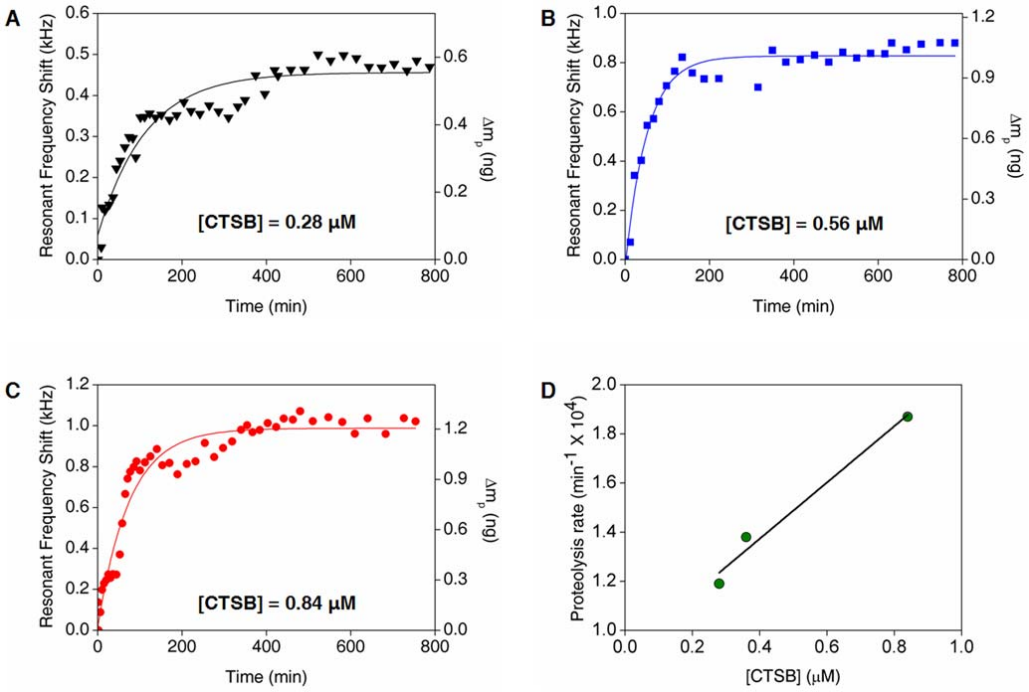
*In situ* resonant frequency shifts, which are measured in buffer solution, attributed to proteolysis of tetrapeptide GFLG are shown with three different CTSB concentrations; (A) [CTSB] = 0.28 µM, (B) [CTSB] = 0.56 µM, and (C) [CTSB] = 0.84 µM. Further, the mass of cleft peptides driven by protease (with a given protease concentration) is computed from *in situ* resonant frequency shift measured in buffer solution due to proteolysis. These resonant frequency shifts with respect to time are well fitted with the Langmuir kinetic model, which allows for extraction of rate constant for proteolysis, *k_p_*. (D) The rate constant for proteolysis, *k_p_*, extracted from *in situ* resonant frequency shift is shown to be linearly proportional to CTSB concentrations, [CTSB]. This indicates that the proteolysis can be controlled by enzyme concentrations, which will be related to drug design.

For quantitative characterization of the kinetics of proteolysis of peptides driven by protease, we take into account the nanomechanical, *in situ* monitoring of proteolysis in a real-time using resonant microcantilever, whose surface was functionalized by PEG-GFLG chains, immersed in buffer solution. Figure 3A-C shows the resonant frequency shifts, which were measured in buffer solution in a real-time after injection of CTSB, for three different cantilevers in response to proteolysis by protease with three different concentrations, i.e. [CTSB] = 0.28 µM, 0.56 µM, and 0.84 µM, respectively. Here, for *in situ* bioassay, we have employed the three different cantilevers, which was functionalized with peptide chains, and their resonant frequencies in buffer solution are 111.452 kHz (for [CTSB] = 0.28 µM), 111.235 kHz (for [CTSB] = 0.56 µM), and 112.196 kHz (for [CTSB] = 0.84 µM), respectively. Since the total mass of cleft peptides can be measured from the resonant frequency shift measured in dry air due to proteolysis, the change of hydrodynamic loading due to proteolysis can be also estimated from the resonant frequency shift evaluated in buffer solution due to proteolysis (see Eqs. (3) and (4) and also Ref. [19]). It is shown that the change of hydrodynamic loading is comparable to the mass of cleft peptides, i.e. Δ*M_h_* ≈ Δ*m_P_* (see above). Thus, the total mass of cleft peptides in a real-time can be obtained from the *in situ* resonant frequency shift measured in buffer solution due to proteolysis (protease) in a real-time such as Δ*m_P_*(*t*) = (*M_c_* + *M_h_*)(Δ*ω_P_^L^*(*t*)/*ω_L_*) (see Eqs. 3 and 4), where Δ*m_P_*(*t*), Δ*ω_P_^L^*(*t*), and *ω_L_* are the total mass of cleft peptides, the *in situ* resonant frequency shift estimated in buffer solution due to proteolysis, and the resonant frequency of a cantilever, which was functionalized by GFLG-PEG chains, immersed in buffer solution, respectively. *M_c_* is the mass of a cantilever, and *M_h_* is the hydrodynamic loading, which can be computed from Eq. 2.

Here, the Langmuir kinetic model has been revisited in order to understand the kinetics of proteolysis dictated by resonant frequency shift measured in buffer solution due to such a proteolysis. Langmuir kinetic model [20], [34] demonstrates the rate equation for dissociation of molecules (proteolysis of peptides) on the surface.
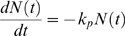
(5)


where *N*(*t*) is the number of peptide chains immobilized on the surface at time *t*, and *k_p_* is the rate constant for proteolysis of peptide chains at specific CTSB concentration in buffer solution. Here, it is assumed that proteolysis event is irreversible process, that is, proteolyzed peptides (i.e. PEG-GF peptide) cannot be specifically bound to cleft peptides (i.e. LG sequence) on a cantilever surface. Such kinetic model provides that *N*(*t*) = *N*_0_exp(–*k_p_t*), where *N*_0_ is the number of peptide chains immobilized on a cantilever surface at initial time (before injection of solution containing CTSB). Consequently, the number of proteolyzed peptide chains, is given by Δ*N_p_*(*t*) = *N*_0_[1 – exp(–*k_p_t*)]. Accordingly, since the total mass of proteolyzed peptides can be obtained from the number of proteolyzed peptides by multiplication of molecular mass (weight), the total mass of proteolyzed peptides is represented in the form of Δ*m_P_*(*t*) = Δ*m_P_* ^0^[1 – exp(–*k_p_t*)], where Δ*m_P_* ^0^ is the total mass of proteolyzed peptides at final state. Since we have a relation of Δ*m_P_*(*t*) = (*M_c_* + *M_h_*)( Δ*ω_P_^L^*(*t*)/*ω_L_*) (for details, see above), the resonant frequency shift measured in buffer solution in a real-time driven by proteolysis is given as Δ*ω_P_^L^*(*t*) = Δ*ω_P_* ^0^[1 – exp(–*k_p_t*)], where Δ*ω_P_* ^0^ is the steady-state value of frequency shift measured in buffer solution due to proteolysis. Here, we have a relation of Δ*m_P_* ^0^ = (*M_c_* + *M_h_*)(Δ*ω_P_* ^0^/*ω_L_*). It is remarkably shown that the *in situ* resonant frequency shift estimated in buffer solution arising from proteolysis in a real-time is well fitted with the Langmuir kinetic model (see Figure 3A-C). Herein, the proteolysis rate, *k_p_*, at CTSB concentration of 0.28 µM in buffer solution is computed as *k_p_* = 1.19×10^4^ min^−1^, and the rate constant for proteolysis, *k_p_*, increases with respect to CTSB concentration (Figure 3D). Moreover, the increase rate of proteolysis, *R* ≡ *dk_p_*/*d*[CTSB], with respect to CTSB concentration, [CTSB], is evaluated as *R* = 1.15 µM^−1^·min^−1^. This indicates that proteolysis rate, *k_p_*, can be controlled by concentration of protease. Further, the deviation of experimental data of *in situ* frequency shift measured in buffer solution from the kinetic model may be ascribed to low quality factor of microcantilever immersed in buffer solution (e.g. *Q* = ∼4 in buffer solution). Conclusively, our result suggests that the proteolysis of peptides and its related kinetics can be depicted by nanomechanical biosensors such as resonant microcantilever sensors.

### Conclusion

We have first demonstrated the nanomechanical, *in situ* monitoring of proteolysis of tetrapeptide GFLG (GlyPheLysGly) induced by cystein protease, Cathepsin B (CTSB), using resonant microcantilever immersed in buffer solution. It is shown that resonant microcantilever enables us to quantify the amount of proteolyzed peptide chains, and consequently, the proteolysis efficiency with respect to protease concentration. Moreover, it is very remarkable that the *in situ* resonant frequency shift, measured in buffer solution, in response to proteolysis is well described by Langmuir kinetic model, and that proteolysis rate increases linearly with respect to protease concentration. This indicates that resonant microcantilever allows for the comprehensive characterization of the kinetics of peptide-protease interactions, which is essential for understanding of the mechanism of disease development and their treatment by drug. In the long run, it is implied that the resonant microcantilever may enable not only the label-free detection of biochemical reaction, shedding light on the early diagnosis of disease, such as proteolysis but also the quantitative understanding of the kinetics of biochemical reaction, related to smart drug design.

### Supporting Information

The schematic illustration of synthesis of tetrapeptide conjugated to PEG is demonstrated. The supplementary results such as AFM imaging of functionalized cantilevers (with or without exposure to protease) and MALDI-TOF mass spectrometry (for GFLG-PEG chain as well as cleft chain) are presented. Also, the control experiments for cantilever assay as well as measurement of frequency shift in dry air due to proteolysis are provided.

## Supporting Information

Figure S1 Figure S1. (A) Schematic illustration of synthesis of PEG-COOH and PEG-GFLG-COOH (for details, see Methods and Materials), and (B) FT-IR spectra of PEG-OH and PEG-COOH. FT-IR spectra confirms the synthesis of PEG-COOH
(3.21 MB TIF)

Figure S2 Matrix Assisted Laser Desorption/Ionization - Time Of Flight (MALDI-TOF) mass spectrometry of (A) PEG-COOH, (B) PEG-GFLG, and (C) cleft peptides (PEG-GF) induced by protease. It is shown that molecular mass of a single GFLG-PEG is 8.64 zg (zepto-gram = 10^−21^ g), while molecular mass of a cleft peptide by protease is 8.30 zg. Here, the molecular mass of a single PEG is 8.30 zg. This indicates that protease specifically cleaves the peptide sequence GFLG.
(6.58 MB TIF)

Figure S3 The resonances were measured in a dry air for a bare cantilever (black solid line) for Cantilever 1 in Table 1, a cantilever functionalized by PEG-GFLG chains (blue solid lines; with concentration of 10 mM), and such a functionalized cantilever in exposure to protease (CTSB with concentration of 0.28 µM), respectively. It is shown that peptide immobilization reduces the resonance of a cantilever (due to increase of overall mass) whereas proteolysis of peptides (by protease) increases the resonance of a cantilever (arising from decrease of overall mass).
(3.53 MB TIF)

Figure S4 Negative control experiments: Resonance behaviors of (A) cantilever, which is functionalized by PEG-GFLG chains, in buffer solution which does not contain protease, (B) a bare cantilever in buffer solution containing protease, and (C) cantilever, which is functionalized by PEG, in buffer solution including protease. These negative control experiments have proved that effect of shaking of buffer solution by resonant cantilever is ignorable, that non-specific binding of CTSB into a cantilever is unlikely to occur, and that protease specifically cleave the GFLG rather than PEG.
(5.01 MB TIF)

Figure S5 AFM images of (A) the surface of a bare cantilever, (B) the surface of a cantilever functionalized by PEG-GFLG chains, and (C) the surface of a cantilever (functionalized by PEG-GFLG chains) in exposure to protease (Cathepsin B), respectively, are shown. As shown in AFM images, peptide immobilization increases the surface roughness of a cantilever, while proteolysis of peptides by protease decreases the surface roughness. This confirms the proteolysis events. (D) For quantitative comparison, we introduce the average height *H*, which indicates the surface roughness, such as *H* = (1/*L*_1_ *L*_2_)∫∫*h*(*x*,*y*)*dxdy*. Here, *h*(*x*,*y*) represents a height of a point (x, y) in the scanned area with a dimension of *L*_1_×*L*_2_ (where *L*_1_ = *L*_2_ = 10 µm). It should be noted that average height *H* presents the quantity for surface roughness rather than actual height. It is shown that peptide immobilization increases the surface roughness of a cantilever enormously, whereas the proteolysis reduces the surface roughness. However, the proteolysis by protease does not reduce the surface roughness as much as the surface of a bare cantilever. This confirms the specific proteolysis of GFLG.
(4.07 MB TIF)
