# Supplementary figures and images for: Nanomechanical In Situ Monitoring of Proteolysis of Peptide by Cathepsin B
Source: PLoS One. 2009 Jul 16;4(7):e6248. doi: 10.1371/journal.pone.0006248 (PMC2707113; doi:10.1371/journal.pone.0006248)

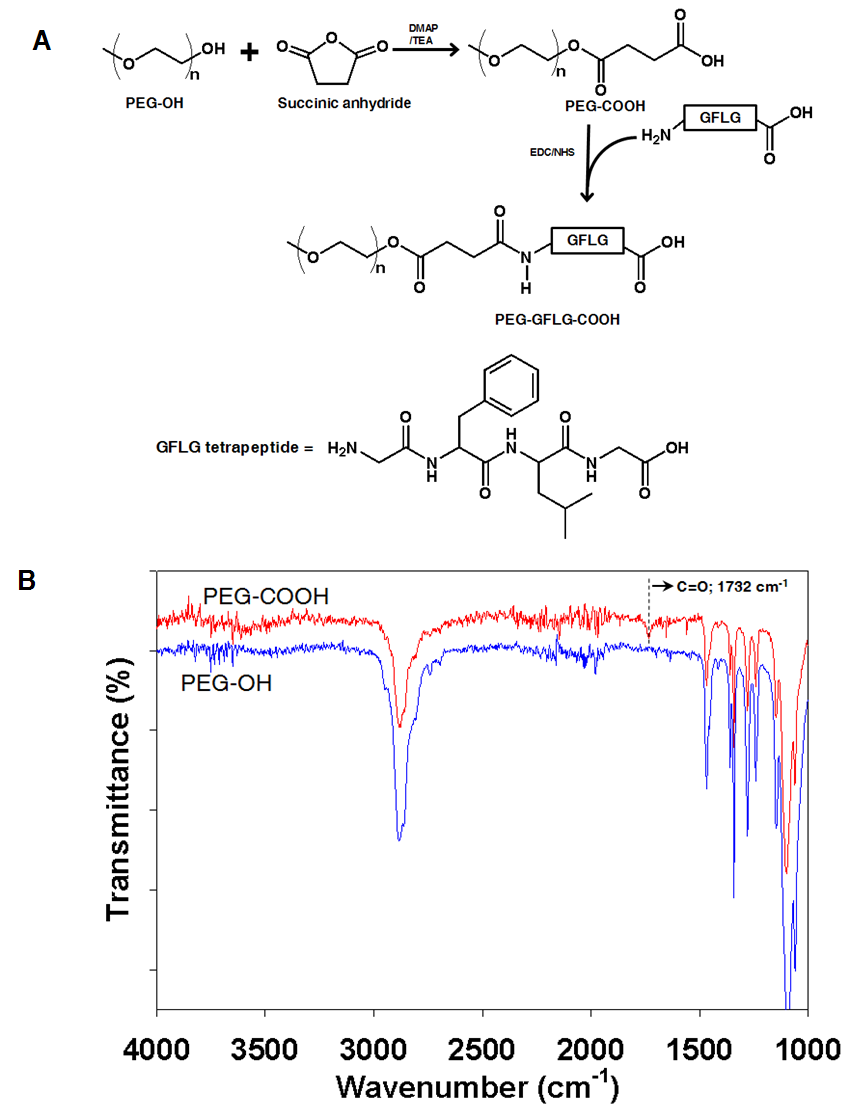

Supplement: Figure S1 — (A) Schematic illustration of synthesis of PEG-COOH and PEG-GFLG-COOH (for details, see Methods and Materials), and (B) FT-IR spectra of PEG-OH and PEG-COOH. FT-IR spectra confirms the synthesis of PEG-COOH (3.21 MB TIF) [file pone.0006248.s001.tif]

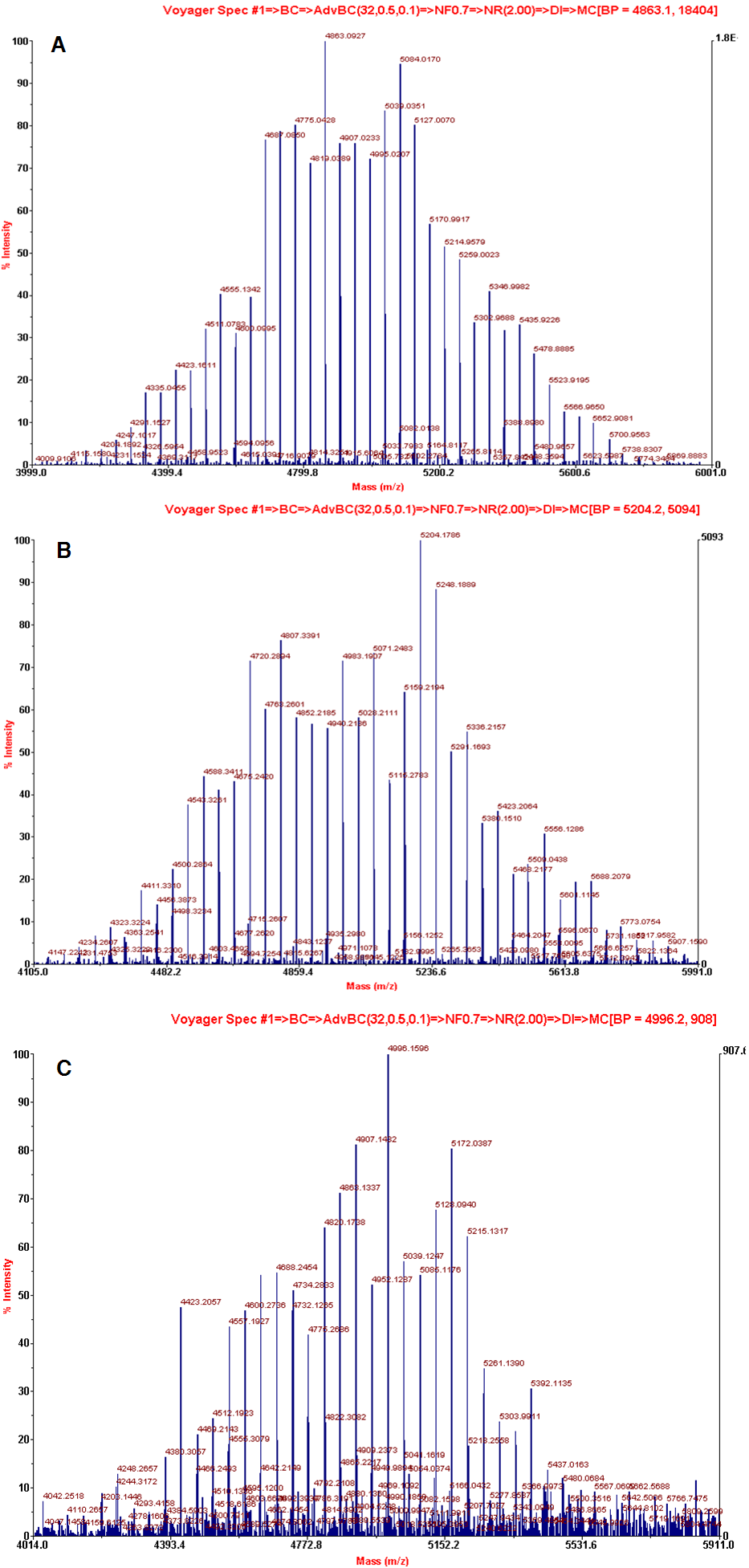

Supplement: Figure S2 — Matrix Assisted Laser Desorption/Ionization - Time Of Flight (MALDI-TOF) mass spectrometry of (A) PEG-COOH, (B) PEG-GFLG, and (C) cleft peptides (PEG-GF) induced by protease. It is shown that molecular mass of a single GFLG-PEG is 8.64 zg (zepto-gram = 10−21 g), while molecular mass of a cleft peptide by protease is 8.30 zg. Here, the molecular mass of a single PEG is 8.30 zg. This indicates that protease specifically cleaves the peptide sequence GFLG. (6.58 MB TIF) [file pone.0006248.s002.tif]

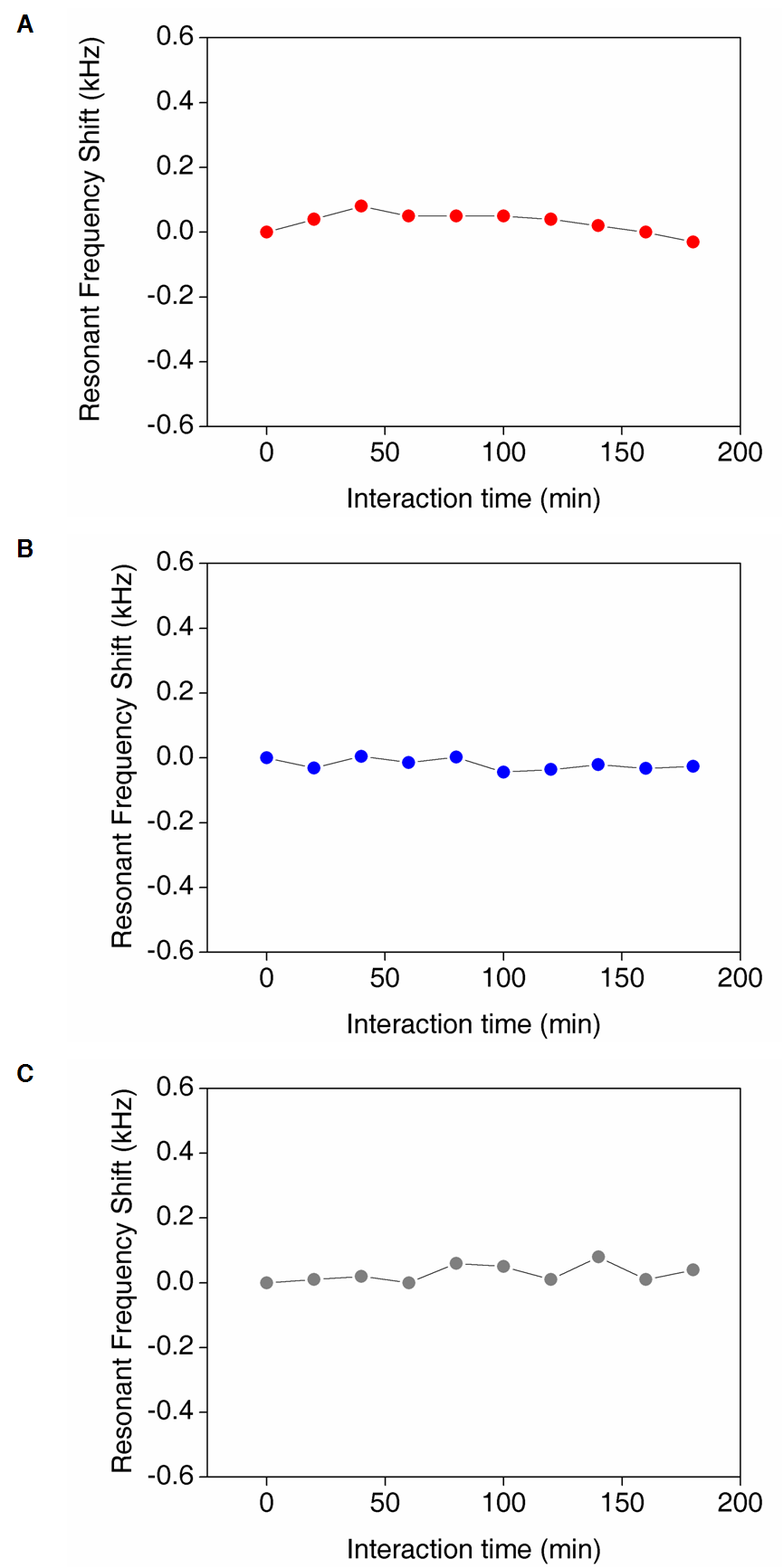

Supplement: Figure S4 — Negative control experiments: Resonance behaviors of (A) cantilever, which is functionalized by PEG-GFLG chains, in buffer solution which does not contain protease, (B) a bare cantilever in buffer solution containing protease, and (C) cantilever, which is functionalized by PEG, in buffer solution including protease. These negative control experiments have proved that effect of shaking of buffer solution by resonant cantilever is ignorable, that non-specific binding of CTSB into a cantilever is unlikely to occur, and that protease specifically cleave the GFLG rather than PEG. (5.01 MB TIF) [file pone.0006248.s004.tif]

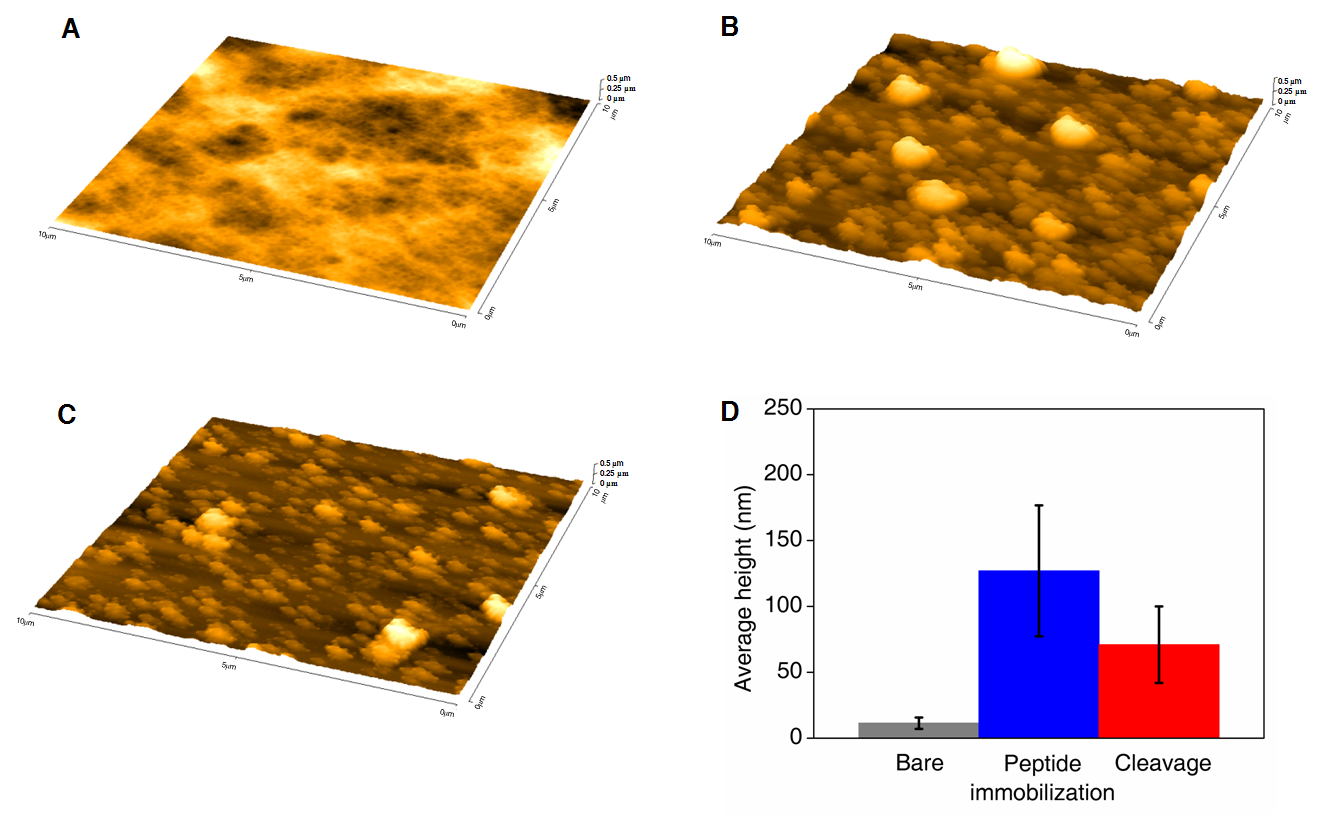

Supplement: Figure S5 — AFM images of (A) the surface of a bare cantilever, (B) the surface of a cantilever functionalized by PEG-GFLG chains, and (C) the surface of a cantilever (functionalized by PEG-GFLG chains) in exposure to protease (Cathepsin B), respectively, are shown. As shown in AFM images, peptide immobilization increases the surface roughness of a cantilever, while proteolysis of peptides by protease decreases the surface roughness. This confirms the proteolysis events. (D) For quantitative comparison, we introduce the average height H, which indicates the surface roughness, such as H = (1/L 1 L 2)∫∫h(x,y)dxdy. Here, h(x,y) represents a height of a point (x, y) in the scanned area with a dimension of L 1×L 2 (where L 1 = L 2 = 10 µm). It should be noted that average height H presents the quantity for surface roughness rather than actual height. It is shown that peptide immobilization increases the surface roughness of a cantilever enormously, whereas the proteolysis reduces the surface roughness. However, the proteolysis by protease does not reduce the surface roughness as much as the surface of a bare cantilever. This confirms the specific proteolysis of GFLG. (4.07 MB TIF) [file pone.0006248.s005.tif]
